# 疑似胸腺瘤的前纵隔肿物胸部CT报告标准

**DOI:** 10.3779/j.issn.1009-3419.2014.02.04

**Published:** 2014-02-20

**Authors:** Edith M. Marom, Melissa L. Rosado-de-Christenson, John F. Bruzzi, Masaki Hara, Joshua R. Sonett, Loren Ketai, 北京大学肿瘤医院 付浩

**Affiliations:** 1 Department of Radiology, The University of Texas, M. D. Anderson Cancer Center, Houston, Texas; 2 University of MissouriKansas City, Kansas City, Missouri; Department of Radiology, Uniformed Services University, Bethesda, Maryland; 3 Department of Radiology, Galway University Hospitals (GUH), Galway, Ireland; 4 Department of Radiology, Nagoya City University Graduate School of Medical Sciences, Nagoya, Japan; 5 Thoracic Surgery Department, Columbia University New York-Presby-terian Hospital, New York, New York;; 6 Department of Radiology, University of New Mexico Health Science Center, Albuquerque, New Mexico

临床医生和放射诊断科医生对影像学的格式化报告和标准术语的要求正在不断增加。一项研究^[1]^发现，CT报告在对肺结节边缘和钙化表述上不统一。另一项研究^[2]^比较了Fleischner Society Glossary中列举的影像术语和它们常用医学词典中的一致性，包括国际疾病分类、标准参考医学术语和统一医学语言系统等，发现Fleischner术语应用率低，仅为3%-36%^[3]^。另一方面，研究提示对特殊疾病的标准影像学报告，如乳腺癌筛查报告可以改善患者治疗结果。因此希望对疑似胸腺瘤的前纵隔肿瘤制定标准的描述术语和提高这些术语的应用率，达到能促进临床医生和放射科医生之间的交流的目的，最终给患者治疗带来获益。除了制定统一的术语，报告中还应包含相关疾病知识，使之具有疾病特异性。美国放射医师协会制定了乳腺影像报告和数据系统，描述在钼靶和超声影像发现的乳腺病灶，发现影像报告结果与组织学结果具有相关性^[4]^。乳腺影像报告和数据系统提供一个对应各种病理类型的百分概率，并在临床上得到广泛应用^[5]^。一个相似的系统应用于甲状腺结节的超声评估，将这些结节分为高度恶性可能和低度恶性可能，指导适当的临床治疗，这个系统被称为甲状腺影像报告数据系统^[6]^。

尽管初步证据表明胸腺瘤特殊的影像学发现对分期或预后很重要，但需要前瞻性数据进一步证实，制定格式化影像报告有利于前瞻性研究。可疑胸腺瘤的前纵隔肿物的CT报告应该遵循以下原则，典型影像表现需要清楚定义和统一描述；相关的阴性表现也应描述。使用被临床医生所接受和应用的一致的描述术语将帮助建立统一的报告语言，以帮助他们对胸腺瘤患者做出治疗决策和进行深入研究。为此，国际胸腺肿瘤协作组织（International Thymic Malignancy Interest Group, ITMIG）核心工作组回顾了现有文献统一的或可能统一的胸部影像学诊断标准，并起草了标准术语及其定义，于2010年11月16日由ITMIG定义与术语拓展组重新提炼，经由ITMIG全体成员的讨论后，于2011年2月获得通过并批准。

## 1 文献回顾

Masaoka分期系统及其改良版是公认与预后有良好相关性的分期系统^[7-10]^，但该分期常于术后才确定，然而，治疗决策要求术前就要明确分期，如局部晚期的胸腺瘤患者可能会接受术前化疗从而获得有效切除^[9, 11]^，如果可以完整切除，甚至是进展期疾病也可以改善生存^[7, 12]^。目前，认为Ⅲ期及Ⅳ期胸腺瘤患者需接受术前化疗^[13-18]^。过去，纵隔影像对于胸腺瘤患者分期价值有限，这可能是因为影像技术落后和胸腺瘤少见的关系。这些因素也影响到胸腺瘤影像学论文的发表和研究的数量，降低了这些研究的统计学效能。CT是目前用于初步评估和随访胸腺瘤患者首选的影像学检查方法^[19]^。在过去10年中，CT技术在常规快速获取薄层断面并重建为高质量图像上取得了飞跃发展。这一结果提高了肿瘤的影像质量、可评估病灶内部情况及肿瘤与周围结构的关系，表明某些CT特征与肿瘤恶性生物学行为和较晚的分期相关。仅有2项研究显示CT表现与Masaoka分期相关^[20, 21, 21a]^。其一包括50例胸腺瘤患者^[21, 21a]^，发现与早期患者相比侵袭性胸腺瘤体积更大，肿瘤有低密度区、钙化、分叶、且边缘不规则。其二，包括99例胸腺瘤患者^[20]^，发现多种征象与进展期胸腺瘤（Ⅲ期、Ⅳ期）相关：体积大、分叶状、不均质低密度区、钙化、浸润周围纵隔脂肪，肿瘤与纵隔结构接触大于等于50%、相邻肺异常、胸腔积液等。然而，多因素分析后显示，仅有体积大，分叶状，浸润周围纵隔脂肪这些影像学特征与更高的疾病分期（Ⅲ期或Ⅳ期）相关^[20]^。

胸腺瘤的组织学分期由世界卫生组织（World Health Organization, WHO）制定。虽然WHO分类在临床应用上因其缺乏足够的可重复性和临床预测价值而存在争议，但研究^[10]^发现CT表现与WHO组织学分类相关。两项研究^[22, 23]^分别评估了45例和76例胸腺瘤患者，发现分叶状、边缘不规则与肿瘤高侵袭性相关，虽然在第3个评估了48例胸腺瘤患者的研究中未得到证实^[24]^。事实上，一些临床医师已用肿瘤体积大、分叶状、肿瘤的均一与否作为患者是否应在术前接受化疗的指标。然而，亟需更大样本开展胸腺瘤影像学表现与生物学行为相关性的大型研究。这些研究应进行国际合作，因为尽管胸腺瘤是前纵隔最常见的原发性肿瘤，但它仅占成人恶性肿瘤不到1%的比率。

## 2 胸腺瘤CT表现描述的建议

每个疑似胸腺瘤的纵隔肿物或诊断为胸腺瘤的CT报告应包括关于原发肿物与周围结构的以下数据，病变的位置和大小，包括x、y、z轴；描述病变的边缘（光滑或分叶状）；是否存在不均质低密度区、钙化、周围脂肪间隙，肿物与周围纵隔组织接触是否大于等于50%，是否直接侵入血管腔。还需包括周围结构的以下信息：膈肌抬高（膈神经受累），相邻肺组织异常，胸腔积液，胸膜结节，淋巴结肿大，及发现远处转移（如肺、肝、肾上腺或腹膜结节）。如果前瞻性的坚持记录这些变量，它们可以在将来的格式化报告中创建一个表格或下拉式菜单，见表 1和表 2。此外，如果常规记录胸腺瘤的这些数据，也可以用于回顾性的研究。

**1:** 原发肿瘤CT报告记录 Documentation of primary tumor characteristics

变量	选择菜单
大小（cm)	X轴（轴位最长径）
	y轴（垂直于最长径）
	z轴（竖直径线）
边缘	平滑
	分叶
内部密度	均一
	不均一
	嚢性
钙化	是
	否
周围脂肪浸润	是
	否
肿瘤接触 > 50%的纵隔结构（之间脂肪层消失）	是（注明哪个结构）
	否
肿瘤与其他纵隔结构接触	是（注明）
	否
直接侵犯血管腔	是（注明血管名称）
	否
注：本表得到版权所有者© 2011 by the International Association for the Study of Lung Cancer复制许可。	

**2:** 侵犯周围结构记录 Documentation of involvement of surrounding structures

变量	选择菜单
邻近肺组织异常	是
	否
胸腔积液	单侧
	双侧
	否
胸膜结节	否
	单侧/双侧
	1个
	2个-5个
	> 5个
纵隔淋巴结肿大（CT轴位短径 > 1 cm)	是（按淋巴结分组注明位置）
	否
紧邻膈神经走行区域	是
	否
膈肌抬高	是
	否
肺结节	是
	否
胸外疑似转移	是（注明部位）
	否
注：本表得到版权所有者© 2011 by the International Association for the Study of Lung Cancer复制许可。	

## 3 胸腺瘤CT报告术语的定义

### 3.1 原发瘤大小

我们建议记录三个轴的肿瘤径线，与胸腺瘤切除后病理报告相一致。横断面选择测量肿瘤的最大径。短轴是同一层面与长径垂直的径线（图 1）。由于肿瘤的方位有时并不完全符合严格的矢状面与冠状面重建，肿瘤的上下径应该是CT窗位肿瘤的上极减去肿瘤的下极（图 1）。

**1:**
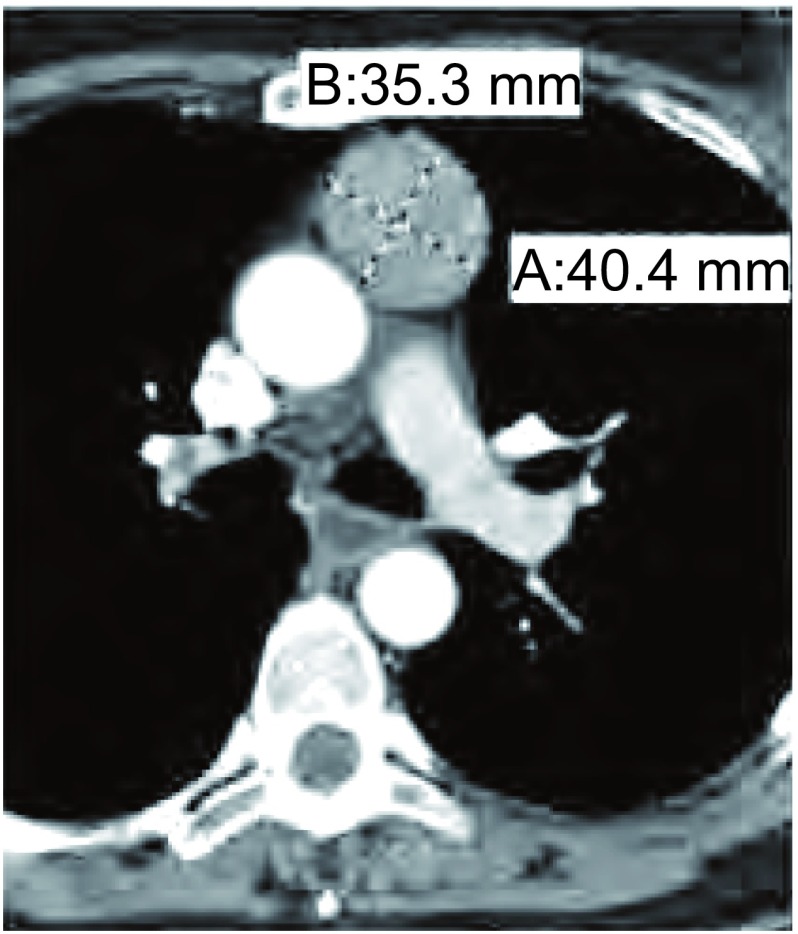
肿瘤大小测量。轴位测量肿瘤的最大径；该病例的最大径线为4 cm。垂直短径为3. 5 cm。肿瘤上极和下极的距离范围为197 mm -149 mm（197-149=48 mm），因此肿瘤上下径为4.8 cm。肿瘤大小应记录为4.0 cm ×3.5 cm×4.8 cm。 Tumor size measurement. The measurement is obtained on the axial slice that demonstrates the tumor's largest diameter; in this case, the largest diameter is 4 cm. The short axis perpendicular to it is 3.5 cm. Tumor ranged in superior to inferior orientation from bed position 197 to 149 (197-149=48 mm) and thus the superior to inferior dimension is 4.8 cm. Thus, tumor size should be reported as 4.0 cm× 3.5 cm×4.8 cm, with the last measurement representing the superior to inferior dimension.

### 3.2 位置

肿瘤多位于前纵隔血管前。部分病变为单侧，某些肿瘤也可越过中线，累及双侧纵隔。

### 3.3 边界

其边缘可以为光滑无毛刺、界线不清或分叶状。光滑的病灶通常呈球形或卵圆形，病灶边界也可以与邻近纵隔形状一致。分叶状表现为一个或多个分叶，边缘凹凸不平（图 2）。

**2:**
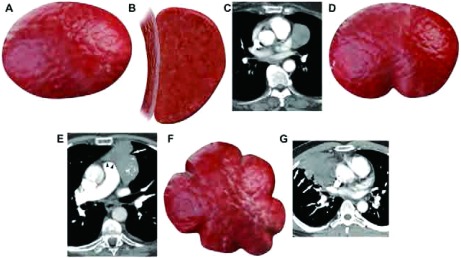
肿瘤边缘形态的CT举例及图解。A：边缘光滑。B：肿瘤边缘光滑，与接触的纵隔结构形态一致。C：增强CT表现肿瘤边缘与接触的纵隔结构形态一致。D：单个切迹的分叶状边缘。E：增强CT（软组织窗）表现单个切迹的分叶状肿瘤，边缘光滑，与接触的周围纵隔结构形态一致（黑箭头所示）。F：多分叶状肿瘤。G：多分叶状肿瘤侵犯肺实质（白箭头所示）。 Schematic and computed tomography (CT) examples of contour. A, Smooth contour. B, Smooth contour conforming to the mediastinal structure the lesion abuts without evidence of lobulation. C, Contrast-enhanced chest CT (soft tissue window) showing a tumor that conforms to the mediastinal structure it abuts. D, Lobulated contour with single sharp notch. E, Contrast-enhanced chest CT (soft tissue window) showing lobulated tumor due to the lateral sharp notch (white arrow) whereas medially the contour is smooth and conforms to the shape of the adjacent mediastinum (black arrowheads). F, Lobulated contour with multiple lobulations. G, CT image of a multilobulated tumor extending into lung parenchyma (white arrows point at lobulations).

### 3.4 密度

胸腺瘤可表现为均一或不均一的密度。不均一密度往往表现为肿瘤内部低密度，应该用软组织或纵隔窗评估。增强扫描可以更好的呈现肿瘤不均一性，只要没有禁忌均应行增强扫描（图 3）。囊性胸腺瘤表现为内部均一的水样低密度，周围包绕软组织或包膜，囊内可有软组织分隔。囊壁上软组织结节是囊性胸腺瘤的另一特征表现。

**3:**
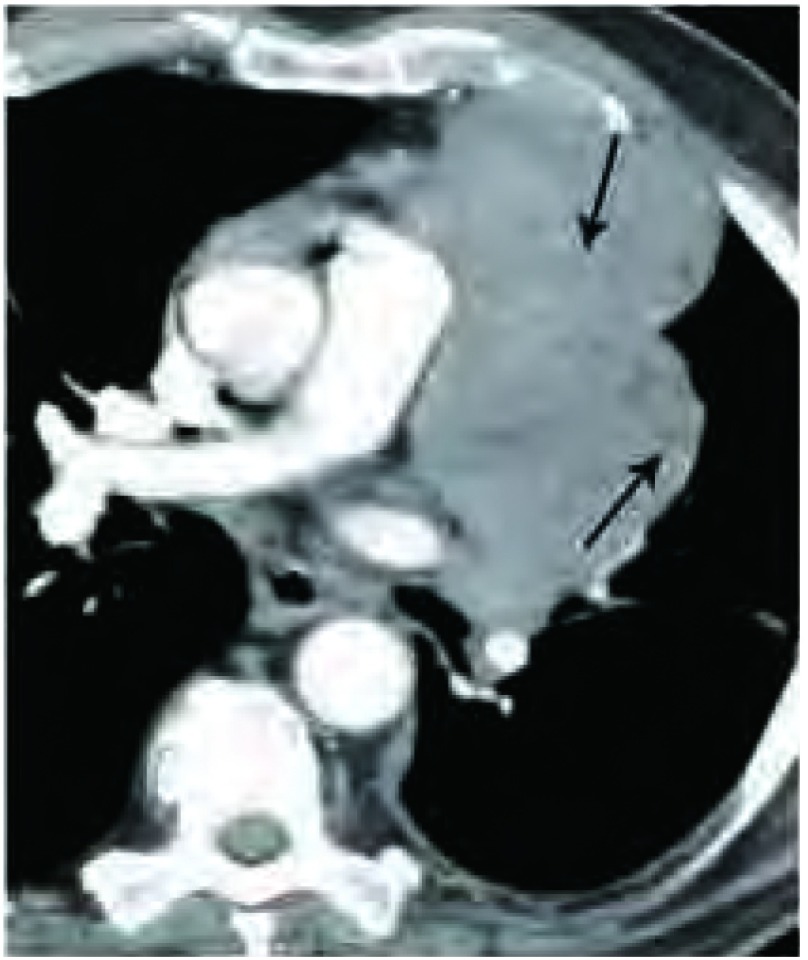
密度。胸部增强CT显示，肺动脉干水平的不均质胸腺瘤。箭头所示为肿瘤中多个低密度区中的两个。 Attenuation. Contrast-enhanced chest computed tomography (CT) (soft tissue window) of a heterogeneous thymoma at the level of the pulmonary trunk. Arrows point to two of the many small low attenuation regions within the mass.

### 3.5 钙化

任何形式的钙化，包括曲线样、点状、或粗颗粒状，都与疾病进展相关。应予以描述。用不同窗位看同一图像，如在骨窗下，可以提高血管内造影剂与肿瘤钙化间的对比差（图 4）。

**4:**
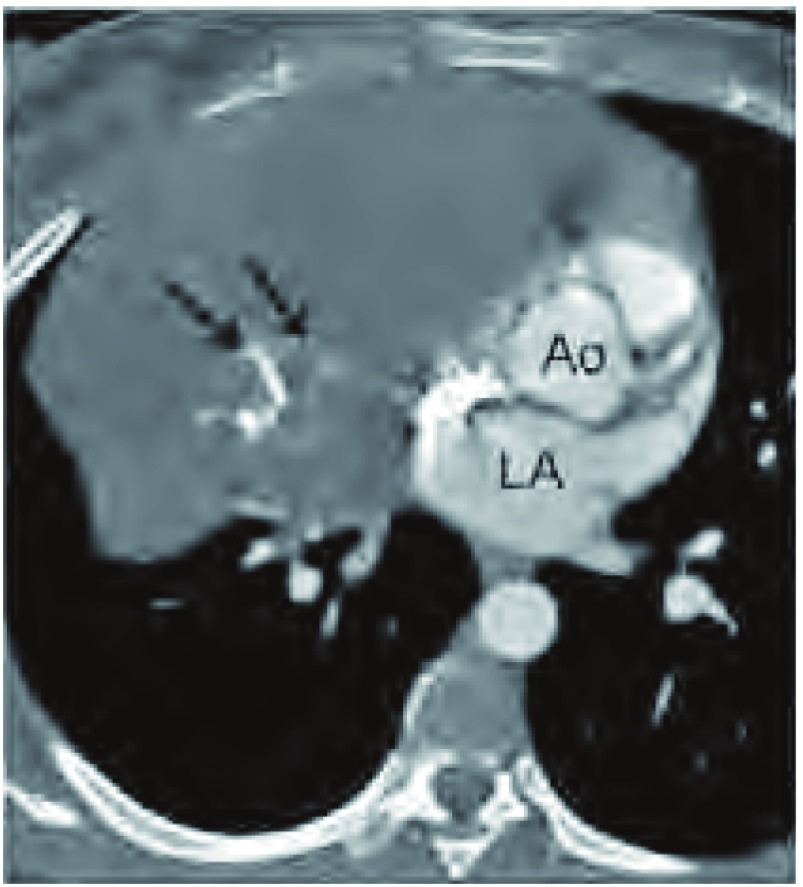
钙化。胸部增强CT（软组织窗）显示，曲线样钙化（黑箭头所示），其形态与高密度的血管形态不同，多数密度高于左心房（LA）和升主动脉（Ao）。 Calcification. Contrast-enhanced chest computed tomography (CT) (soft tissue window) at levels set between bone and mediastinal window. Curvilinear high-density regions (black arrows) are due to calcifications as they do not conform to the expected morphology of an opacified blood vessel, and most are of higher attenuation than opacified blood in the left atrium (LA) and ascending aorta (Ao).

### 3.6 周围纵隔脂肪浸润

只要肿瘤的某一位置上有脂肪浸润，而无需整个肿瘤周围脂肪受累就可定义为肿瘤浸润周围脂肪。这类肿瘤可表现为边界不规则。肿瘤与纵隔血管间无脂肪组织间隙，因为没有看到血管周围纵隔脂肪，不能评价是否有浸润，此时不能定义为周围脂肪浸润（图 5）。

**5:**
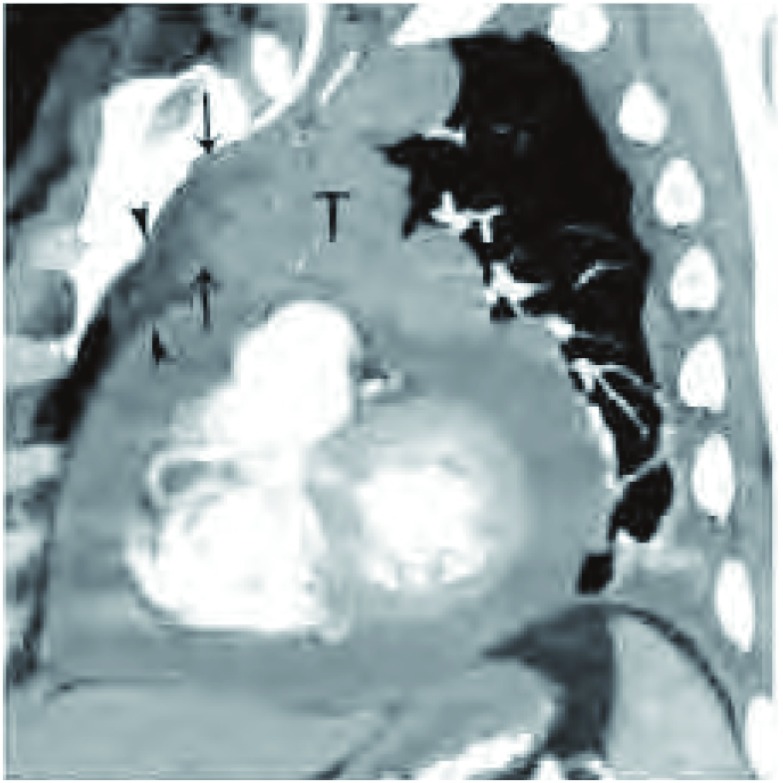
侵犯纵隔脂肪。增强CT冠状位重建（软组织窗）所示，胸腺瘤（T）侵犯纵隔脂肪组织（黑箭头所示）。注意，远离肿瘤的纵隔脂肪密度低于紧邻肿瘤的脂肪密度。 Infiltration of mediastinal fat. Coronal oblique reformatted contrast-enhanced chest computed tomography (CT) (soft tissue window) shows the thymoma (T) with infiltration of surrounding mediastinal fat (black arrows). Notice that the attenuation of the mediastinal fat further away from the tumor (arrowheads) is lower than that immediately adjacent to the lesion.

### 3.7 肿瘤接触≥50%的纵隔结构

为了保持影像报告的一致性，应描述肿物与血管接触面（无脂肪层）占血管环周的百分比（图 6）。

**6:**
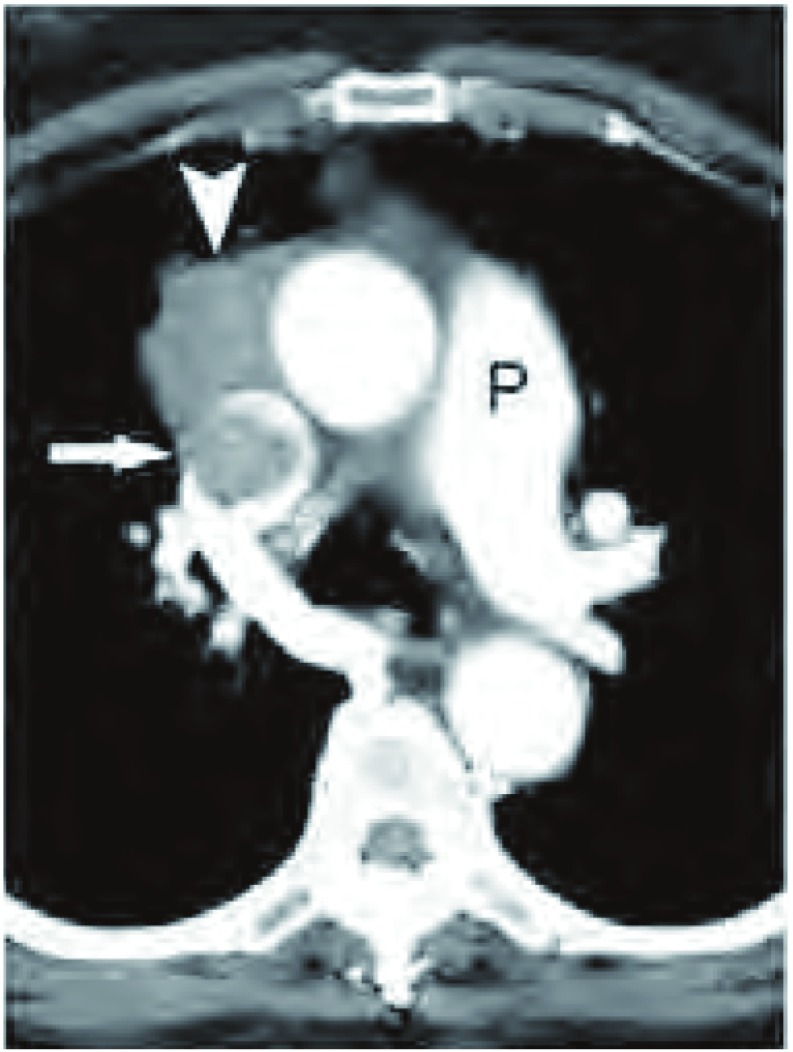
紧邻血管。胸部增强CT（软组织窗）所示，胸腺瘤（T）几乎完全包绕头臂静脉（长箭头所示），而与升主动脉（A）的接触面为30%。短箭头所示为增强的并行静脉。 Abutment of vessels. Contrast-enhanced chest. (CT) (soft tissue window) demonstrates the thymoma (T) nearly completely encasing the right brachiocephalic vein (white arrow), whereas only abutting about 30% of the aorta (A) circumference. Arrowhead: contrast filled collateral vein.

### 3.8 直接侵犯血管

血管受侵很少见，但可表现为直接侵犯至血管腔（图 7）。当出现这种情况时，应描述血管腔的狭窄或畸形。

**7:**
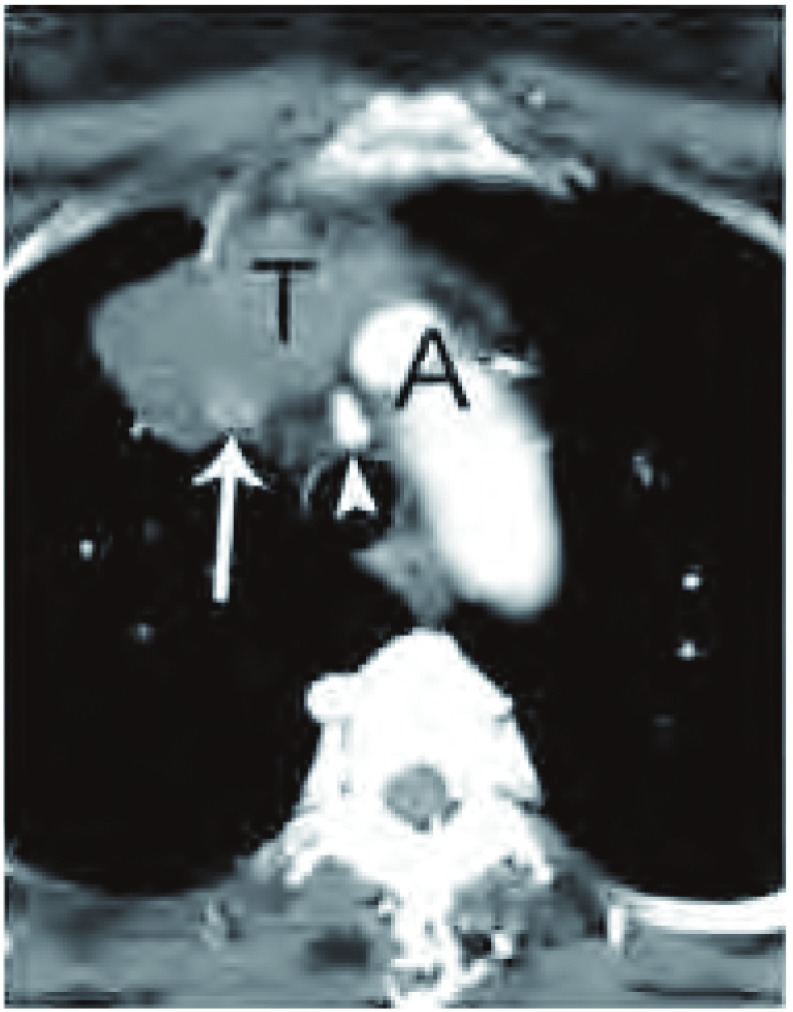
肿瘤侵犯血管。增强胸部CT（软组织窗）示肺动脉干（P）水平，胸腺瘤直接侵犯上腔静脉（长箭头所示）。注意，管腔内肿物软组织密度与原发瘤相似。 Vessel invasion. Contrast- enhanced chest computed tomography (CT) (soft tissue window) at the level of the pulmonary trunk (P) demonstrates a thymoma in the anterior mediastinum (arrowhead) with direct invasion (seen on a more superior slice) into the superior vena cava (white arrow). Note soft tissue attenuation of the intracaval mass similar to that of the primary tumor.

### 3.9 纵隔淋巴结肿大

就如同记录术中清除的所有肿大淋巴结一样，应报告纵隔淋巴结肿大及其位置^[26, 27]^。纵隔淋巴结肿大定义为淋巴结的短径大于1 cm（图 8）。

**8:**
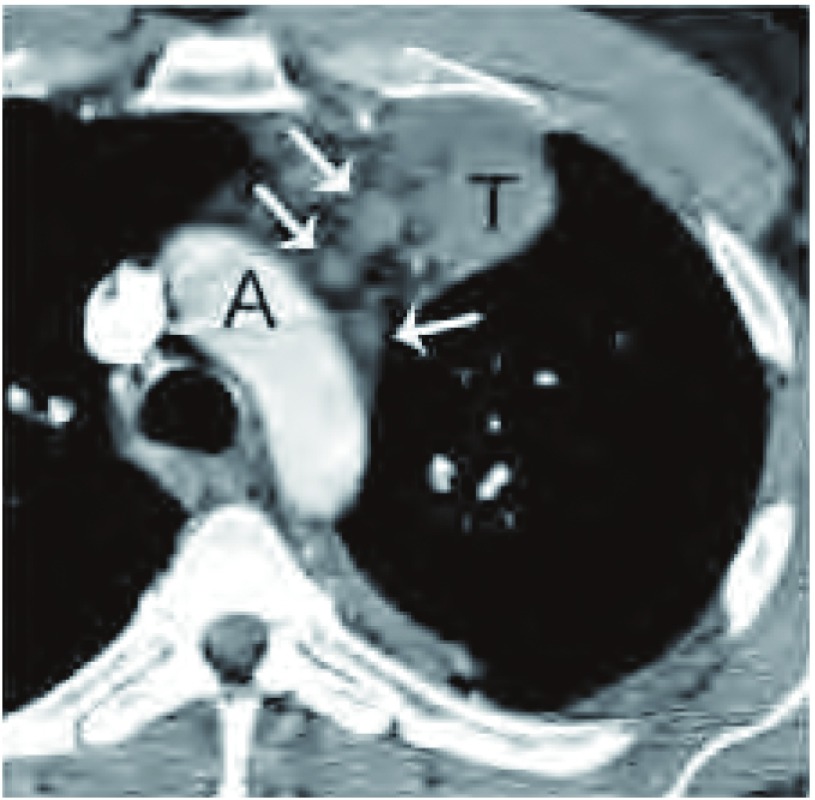
淋巴结肿大。胸部增强CT软组织窗表现，白箭头所示主动脉弓（A）水平胸腺瘤（T）及邻近的肿大淋巴结。术后病理证实这些肿大淋巴结未发现癌。 Lymph node enlargement. Contrast -enhanced chest computed tomography (CT) (soft tissue window) at the level of the aortic arch (A) demonstrates the thymoma (T) and adjacent mildly enlarged ipsilateral mediastinal lymph nodes (white arrows). At surgery, no malignancy was found within these lymph nodes.

### 3.10 相邻肺组织异常

在CT上肿瘤很少表现为直接累及相邻肺组织，多系术中发现。CT最常见的肺组织异常为邻近肿瘤压迫造成的肺不张，但这与肿瘤直接侵犯肺组织很难区分（图 9）。

**9:**
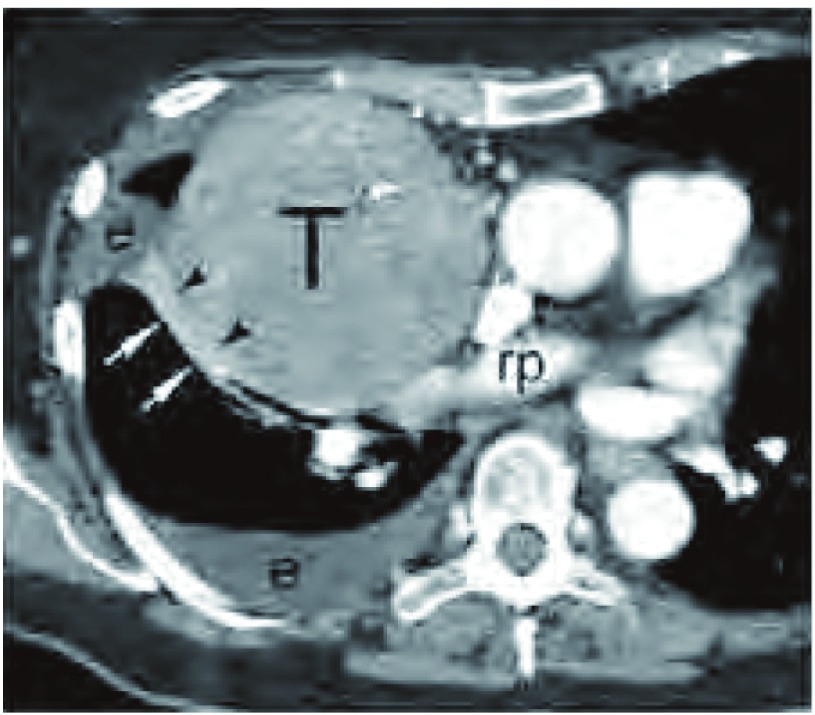
侵犯肺实质。胸部增强CT软组织窗表现，右肺动脉（rp）水平，胸腺瘤压迫右肺中叶支气管，造成中叶肺不张。黑箭头所示不张的肺组织位于肿瘤边缘与右侧叶间裂之间（白箭头所示）。在这个部位很难鉴别肿瘤是否直接侵犯肺实质。注意，有相关的右侧胸腔积液（e）。 Pulmonary parenchymal involvement. Contrast-enhanced chest computed tomography (CT) (soft tissue window), at the level of the right pulmonary artery (rp), shows a thymoma (T) compressing the middle lobe bronchus with resultant atelectasis of the middle lobe. The atelectatic middle lobe is seen between the tumor edge (black arrowheads) and the right major fissure (white arrows). It is difficult to determine whether there is direct tumor invasion into the lung parenchyma at this site. Note the associated right pleural effusion (e).

### 3.11 胸腔积液

胸腺瘤患者出现胸腔积液者并不常见。然而，应报告是否有胸腔积液存在，因胸腔积液常与胸腺癌或其他肿瘤造成的胸膜转移有关（图 9）。

### 3.12 膈肌抬高

包括膈神经在内的手术切除可能会损伤肺功能，并可导致严重的术后并发症^[28]^。术前评估膈神经是否受累的重要性在于，可用膈神经是否受累作为是否需要接受术前新辅助化疗的指标，完整切除肿瘤而不切断膈神经，不仅保留患者肺功能，而且得到生存获益。因此，应报告膈肌是否抬高。此外，还应提及肿瘤邻近膈神经的解剖部位。膈神经从上跨越头臂动脉，行走于锁骨下静脉后，然后横跨肺门前方，右膈神经行走于右心房上的心包，左膈神经行走于左心室上的心包，呈分支状支配肌肉（图 10）。

**10:**
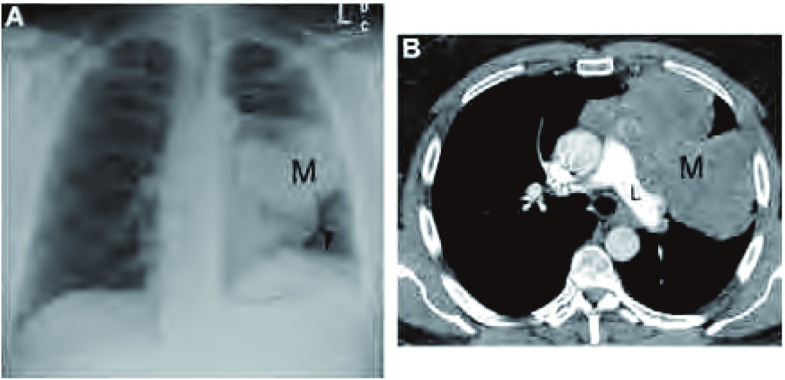
侵犯膈神经。A：后前位胸片显示，巨大的纵隔胸腺瘤（M），伴有左侧的膈肌抬高（箭头所示）。B：胸部增强CT（软组织窗）显示，左肺动脉（L）水平胸腺瘤（M）占据整个左侧肺门，该部位是左侧膈神经的走行区域。 Phrenic nerve involvement. A: Posterior anterior chest radiograph of newly diagnosed thymoma demonstrates a large mediastinal mass (M) with associated elevation of the left hemidiaphragm (arrowhead). B: Contrast-enhanced chest computed tomography (CT) (soft tissue window) at the level of the left pulmonary artery (L) demonstrates the thymoma (M) abutting the entire left side of the mediastinum anterior to the hilum, this location is in the expected course of the left phrenic nerve.

### 3.13 胸膜结节

转移性胸腺瘤常累及胸膜，表现为胸膜软组织结节，范围从小扁豆状结节到大的胸膜肿块，也可发展为结节周围胸膜增厚并累及叶间裂。胸膜转移（Ⅳa期）应区别于肺实质转移（Ⅳb期）。胸膜结节分布于胸膜表面，早期病例通过肺窗能很好评估。而肺实质内结节完全被周围肺实质包围^[29]^（图 11）。

**11:**
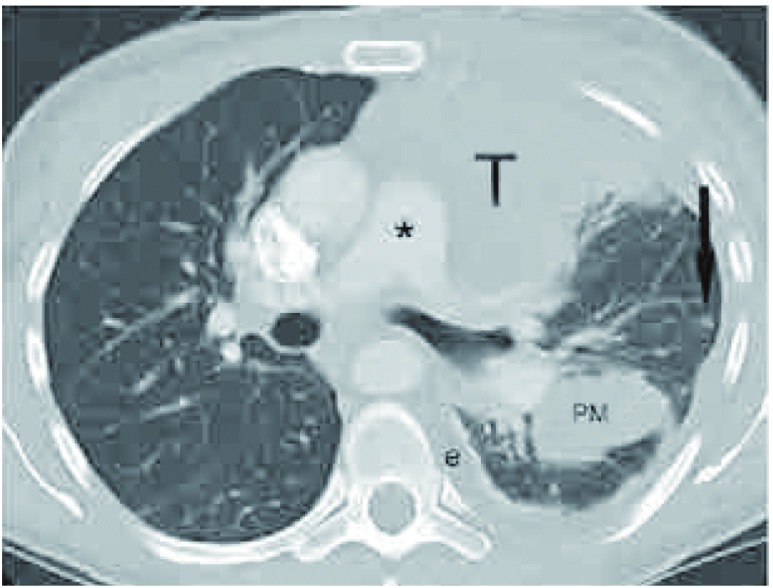
胸膜和肺转移。胸部增强CT（肺窗）显示，胸腺瘤紧邻肺动脉干（*）。左侧叶间裂胸膜转移（PM），左侧胸腔积液（e），和肺实质结节（黑箭头所示）。 Pleural and ulmonary metastasis. Contrast-enhanced chest computed tomography (CT) (lung window)demonstrates the thymoma (T) abutting the pulmonary trunk (*). There is a pleural metastasis within the left major fissure (PM), a left pleural effusion (e), and a pulmonary nodule (black arrow) surrounded by lung.

### 3.14 远处转移

远处转移不常见，为Ⅳb期病变。最常见的转移部位是肺，然后为肝、淋巴结及骨^[30]^。

总之，对怀疑为胸腺瘤的纵隔肿物尤其是晚期胸腺瘤的特征性的影像学表现，应用正确标准的术语描述，这样可增加CT报告的价值，有利于临床医师与放射科医师间的沟通，使放射科医师在帮助临床诊断及术前治疗胸腺瘤患者中起重要作用。
